# Sex-specific effects of 17-β-estradiol and bisphenol A on neutrophil function and phenotype – does centrifugation matter?

**DOI:** 10.1186/s13293-026-00906-9

**Published:** 2026-04-24

**Authors:** Kathrin Fürst, Michael Gruber, Diane Bitzinger, Richard F. Kraus

**Affiliations:** Department of Anesthesiology, University Hospital Regensburg, Franz-Josef-Strauss-Allee 11, 93053 Regensburg, Germany

**Keywords:** Neutrophils, 17-β-estradiol, Bisphenol A, Centrifugation, Sex differences, Flow cytometry, Live-cell imaging

## Abstract

**Background:**

Neutrophils (polymorphonuclear leukocytes, PMNs) have long been underestimated in the context of autoimmunity. To elucidate their role from a sex-specific perspective, an immunoendocrinological framework offers a compelling investigative approach. We hypothesize that the sex hormone 17-β-estradiol (E2) and the endocrine-disrupting chemical bisphenol A (BPA) modulate PMN functions in a sex- and concentration-dependent manner. In addition, we consider the potential confounding effects of centrifugation protocols employed during PMN isolation.

**Methods:**

PMNs from healthy women and men were incubated with different concentrations of E2 (zero; low: 0.01 µM; high: 5µM) and BPA (zero; low: 1.6 µM; high: 16 µM). Migration behavior was assessed using live-cell imaging (*n* = 6 per concentration). Changes in cell surface expression and oxidative burst were quantified by flow cytometry (*n* = 12 per concentration).

**Results:**

Among samples from females, low doses E2 and BPA significantly reduced chemotactic migration. High substance concentrations led to a significant track length increase. Samples from males showed impaired migration after BPA incubation and increased migration behavior in the presence of E2 in both concentrations. Partially altered antigen expression was seen only in samples from females. Hereby, both concentrations of the tested substances caused reduced CD11b expression. Low dose E2 increased CD66b expression, low dose BPA induced higher LOX-1 expression. At the low concentration level, both substances led to an increase in Phorbol 12-myristate 13 acetate-stimulated oxidative burst. For PMNs from women, this also applied to high dose E2.

**Conclusion:**

Different concentrations of the hormone E2 and the endocrine-disrupting chemical BPA modulated neutrophil functions and phenotypes. The effects appeared to be sex-specific, providing a possible explanation for sex-specific immune responses. The data are consistent with the hypothesis that centrifugation impairs PMN functions and showed remaining substance effect despite of centrifugation.

**Supplementary Information:**

The online version contains supplementary material available at 10.1186/s13293-026-00906-9.

## Abstract (plain language summary)

As part of the immune system, neutrophils (polymorphonuclear leukocytes, PMNs) act as the first line of defense. They can destroy pathogens, support other immune cells and help protect the body from infections. The field of immunoendocrinology examines how hormones modulate immune cells like PMNs. Studying these interactions, including the effects of 17-β-estradiol (E2), helps to explain sex-specific differences in immune responses. This knowledge may inform more personalized therapeutic approaches. Endocrine-disrupting chemicals (EDCs) are substances that can interfere with the body’s hormones and may hold relevance in this context. Bisphenol A (BPA), found in certain plastics, is one example known to disrupt hormone function. This study investigates high active concentrations of E2 and BPA on PMN responses to enhance the current understanding of the field. We found that both substances modulated neutrophil functions and phenotypes in a sex- and concentration-specific manner. These results highlight the potential involvement of E2 in influencing PMNs within the context of sex-specific immune responses. The findings additionally emphasize socio-political considerations for minimizing BPA exposure. Several comparative projects investigating PMNs and the tested substances have incorporated centrifugation into their experimental designs. Given the possibility that centrifugation affects PMN functions, this study also aims to contribute methodologically to research in this area. Through a carefully structured setup, we were able to first demonstrate the impact of the substances on non-centrifuged cells. In addition, further investigations provided support for the hypothesis that centrifugation influences PMN functions. Notably, the effect of the substances was observed even after prior centrifugation.

**Supplementary Information:**

The online version contains supplementary material available at 10.1186/s13293-026-00906-9.

## Highlights

High doses of E2 and BPA significantly increased chemotactic migration of female PMNs, while low doses led to a significant track length decrease.

Male PMNs showed impaired migration after BPA incubation and increased migration in the presence of E2 for all tested doses.

E2 and BPA led to a partially altered antigen expression only in samples from females.

Low doses of E2 and BPA increased Phorbol 12-myristate 13 acetate-stimulated oxidative burst in both sexes.

Centrifugation at a ‘g-time’ of 90 kgs does not impair PMNs to such an extent, so that the analyzed substances could no longer exert any effect.

**Supplementary Information:**

The online version contains supplementary material available at 10.1186/s13293-026-00906-9.

## Introduction

As a component of the innate immune system, neutrophil granulocytes (PMNs) play a crucial role in maintaining human health. To eliminate invading microorganisms, for example, these cells can migrate specifically to their site of action, a process known as chemotactic migration. Hereby, the Mac-1 (CD11b/CD18) integrin supports targeted cell movement [1, 2]. Further cell surface antigens involved, are the cell adhesion molecule CD66b, the selectin CD62L and the Lectin-like Oxidized Low-Density Lipoprotein LOX-1 [3–5]. At the lesion site PMNs then use various strategies for pathogen defense, like the release of reactive oxygen species (ROS) [6].

As far as the human immune system is concerned, it has been found, that women’s immune responses differ from those of men. Women are more likely to develop autoimmune diseases [7]. One possible explanation for this could be the different levels of the female sex hormone 17-β-estradiol (E2) in women and men [8]. The field of immunoendocrinology exists to bring the two areas mentioned above - the immune system and the endocrine system - into a common context. Research revealed that various immune cells can not only produce hormones themselves, but can also be influenced by these messengers [9, 10]. For instance, E2 has been demonstrated to delay PMN apoptosis in a dose-dependent manner [8]. Additionally, E2 appears to modulate other PMN functions, including migration and ROS production [11–13].

In addition to endogenous hormones, there are also endocrine-disrupting chemicals (EDCs). These may be xenobiotics or occur naturally, for example in food. Because of their structural similarity to the body’s own hormones, they can affect the human endocrine system. One EDC that has come to the fore in the past is bisphenol A (BPA). This chemical compound is used, for example, in plastic bottles [10]. Like E2, it also has been shown to affect PMN functions [14, 15].

In our study, the concentration- and sex-dependent influences of E2 and BPA on PMN functions (chemotaxis, surface epitope expression, ROS production) were analyzed. In addition, sex-specific differences, and the influence of centrifugation during the isolation procedure were examined.

## Materials and methods

### Blood sampling and cell isolation

Whole blood was collected from healthy, non-pregnant women and men (for detailed participant data see Supplementary Table 1) after informed consent was obtained in accordance with the positive ethics declaration University Regensburg: 22-3070_1-101. Blood was drawn into one 9.0 mL lithium heparin tube and one 7.5 mL serum tube (both from SARSTEDT AG & Co. KG, Nuembrecht, Germany). Cell isolation was performed with Gelafundin^®^ ISO (40 mg/mL; B. Braun SE, Melsungen, Germany) sedimentation as described previously [16–18]. By adding 0.2 mL of Gelafundin^®^ to 1.8 mL of lithium heparin whole blood, a PMN-rich supernatant was obtained after 30 min sedimentation. Both, the supernatant, and the subject serum, were stored at room temperature in separate tubes until the EDC or hormone was added. To receive serum, the whole blood in the serum tube was centrifuged (1180*g*) for 10 min at room temperature.

### Incubation with E2 and BPA

After a 1:1 dilution of the cell supernatant with serum, E2 or BPA was added. Preparation of samples for live-cell imaging was carried out immediately before starting the microscope (see Supplementary Table 2). Samples for flow cytometry analysis were incubated at 37 °C for three hours. E2 (Sigma-Aldrich E8875) was used at a concentration of 5 µM (β-Estradiol [5 µM]) and 0.01 µM (β-Estradiol [0.01 µM]). The third sample was the zero sample without E2. Roswell Park Memorial Institute (RPMI) 1640 medium (PAN Biotech GmbH, Aidenbach, Germany) was added to this sample to analyze equal volumes. The EDC BPA (Supelco 239658) was used at a concentration of 16 µM (BPA [16 µM]) and 1.6 µM (BPA [1.6 µM]). RPMI medium was included in the zero sample as well. To analyze a possible effect of centrifugation and a joint effect of centrifugation and substance, the PMN-rich isolate, except for the zero sample, was centrifuged for 300 s at 300*g* and room temperature in the second series of experiments (see Supplementary Fig. 1b for the experimental setup) [19].

### Live-cell imaging

For microscopy experiments, 3D-µ-chemotaxis slides (Ibidi GmbH, Graefelfing, Germany) were used. These are slides with three individual channels, each bordered on both sides by two reservoirs (see Supplementary Fig. 1a). After filling these channels with 6.5 µL of a gel (composition see Supplementary Fig. 1b) and curing for 30 min at 37 °C and 5% CO_2_, 65 µL of a n-formyl-methionyl-leucyl-phenylalanine (fMLP) (fMLP, Sigma Aldrich, St. Louis, USA) - serum mixture was filled into all left reservoirs. The right reservoirs were filled with 65 µL of the different samples (see corresponding test series Supplementary Fig. 1). Immediately after completion, images were taken under the microscope. To observe migration behavior, PMNs were observed for approximately 22 h using a Leica DMi8 microscope (Leica Microsystems GmbH, Wetzlar, Germany). Phase contrast images were taken every 30 s with the Leica DFC9000 GT camera (Leica Microsystems GmbH, Wetzlar, Germany) (using Leica Application Suite X software, version 3.4.2.18368, Leica GmbH, Wetzlar, Germany). Constant experimental conditions at 37 °C, 50% rel. humidity and 5% CO_2_ were provided by an incubator (ibidi© GmbH, Martinsried, Germany).

The migration behavior was analyzed using Imaris^®^ software (version 9.0.2, Bitplane, Zurich, Switzerland). Every 90 min, a 30-min image section was analyzed. Fixed default settings (XY diameter = 13 μm, quality above 0.847) allowed uniform detection of PMNs based on individual ‘spots’. Six individual parameters were recorded to describe cell migration (see Table 1). The X/Y track displacement describes the movement of the cell in the X or Y direction. While the track displacement length indicates the Euclidean length of the distance travelled (see blue line in Supplementary Fig. 2), the track length (TL) indicates the total distance travelled by the cell within 30 min (see grey line in Supplementary Fig. 2). The track duration provides information about the observation period of a cell, while the track speed mean describes the average speed of movement. Track straightness was also calculated. It was defined as track displacement length/track length.

**Table 1:** Cell migration parameter

Cell migration parameter
Track displacement X/Y [µm]
Track displacement length [µm]
Track length [µm]
Track duration [s]
Track speed mean [µm/s]
Track straightness

The Excel files, which contained all the migration parameters mentioned above, were further analyzed using the SPSS^®^ statistics program (version 28.0.0.0, IBM, Armonk, NY, USA). Only PMNs with a track duration ≥ 900 s and a track length ≥ 25 μm were included in this analysis.

### Flow cytometry

The influence on PMN antigen expression and oxidative burst was analyzed using the FACS-CaliburTM flow cytometer (Becton & Dickinson, Heidelberg, Germany) and CellQuest Pro software (version 5.2, Becton & Dickinson, Bioscience, San Jose, USA). For every sample, two independent preparations were performed, and each was measured separately.

#### Antigen expression measurement

To quantify a possible change in the epitope density of CD11b, CD62L, CD66b and LOX-1, 20 µL of each sample were mixed in duplicate with 1 mL of Dulbecco’s phosphate-buffered saline solution with MgCl_2_ and CaCl_2_ (PBS) (Sigma, D8662). After centrifugation at 4 °C and 1500 rpm (425*g*) for three min, the supernatant was tipped off. In the separate tubes, either no antibody (blank), 2 µL of LOX-1 antibody (Allophycocyanin (APC)-conjugated, REA1188, Miltenyi Biotec B.V. & Co. KG) or 5 µL of the three differently labelled antibodies CD11b (Phycoerythrin (PE)-conjugated, ICRF44, BioLegend, San Diego, CA, USA), CD62L (Fluorescein (FITC)-conjugated, DREG-65, BioLegend) and CD66b (APC-conjugated, G10F5, BioLegend) were added. After incubation for 15 min at 4 °C in the dark, a wash step was performed by adding 2 mL PBS and repeating the centrifugation at constant 'g-time' [17]. After tipping and adding 200 µL PBS, the samples were measured using the different wavelengths.

#### Oxidative burst measurement

To measure ROS release, 20 µL of sample solutions were mixed in duplicate with 1 mL PBS without MgCl_2_ and CaCl_2_ (Sigma, D8537) and 10 µL dihydrorhodamine (DHR) was added. After that, 10 µL of seminaphtharhodafluorine (SNARF, 10 µM, Invitrogen, Eugene, OR, USA) was introduced. Two separate approaches were used for cell stimulation (fMLP or Phorbol 12-myristate 13-acetate (PMA), both 10 µM, Sigma-Aldrich GmbH). Prior to incubation at 37 °C for 10 min, 10 µL tumor necrosis factor α (TNFα, 1 µg/mL, Thermo Fisher Scientific) was added to the fMLP samples. Subsequently, stimulation was performed using fMLP or PMA. After a further incubation for 20 min at 37 °C, dead cells were counterstained with 10 µL Propidium Iodide (PI; 33671, 1.5 mM, Serva Electrophoresis GmbH, Heidelberg, Germany).

#### Data analysis

All measurement data from the flow cytometry experiments were analyzed using the FlowJo™ software (V10.0.7, Becton & Dickinson, Franklin Lakes, NJ, USA). PMNs were initially gated on a forward scatter versus side scatter dot plot and identified by CD66b-positive staining. The various fluorescence channels were then assigned, and the data was further analyzed using Excel. The APC-labelled antibodies (CD66b and LOX-1) were detected in the FL4-H channel. FL2-H showed the fluorescence behavior of the PE-labelled surface marker CD11b, FL1-H was assigned to CD62L (FITC). In the FL3-H fluorescence channel, the dead (PI-labelled) cells were differentiated from the vital cells. All PMNs (FL1-H) could then be selected from the vital cells.

### Statistical analysis

All data collected in Excel were analyzed using the IBM^®^ SPSS^®^ statistical program (version 28.0.0.0, IBM). The Kolmogorov-Smirnov test was employed to assess the normality of the distribution. If this criterion was met, the means of each group were compared using a one-way analysis of variance (ANOVA) and standard deviations were calculated (unless otherwise stated). Homogeneity of variance was evaluated using the Leveneʼs test. Where applicable, post-hoc analysis was performed using the Bonferroniʼs test, otherwise the Dunnett-T3 test was applied. For data that were not normally distributed, the Kruskal-Wallis test was used. These results were then visualized using box plots showing the median, upper, and lower quartiles, interquartile range (IQR), and minimum and maximum values. Non-parametric tests were employed when only some of the measurements in each group were not normally distributed. All p-values ≤ 0.05 were evaluated as statistically significant and marked in the corresponding illustrations.

## Results

### Chemotactic migration

#### The impact of E2 on non-centrifuged cells

**Fig. 1:**
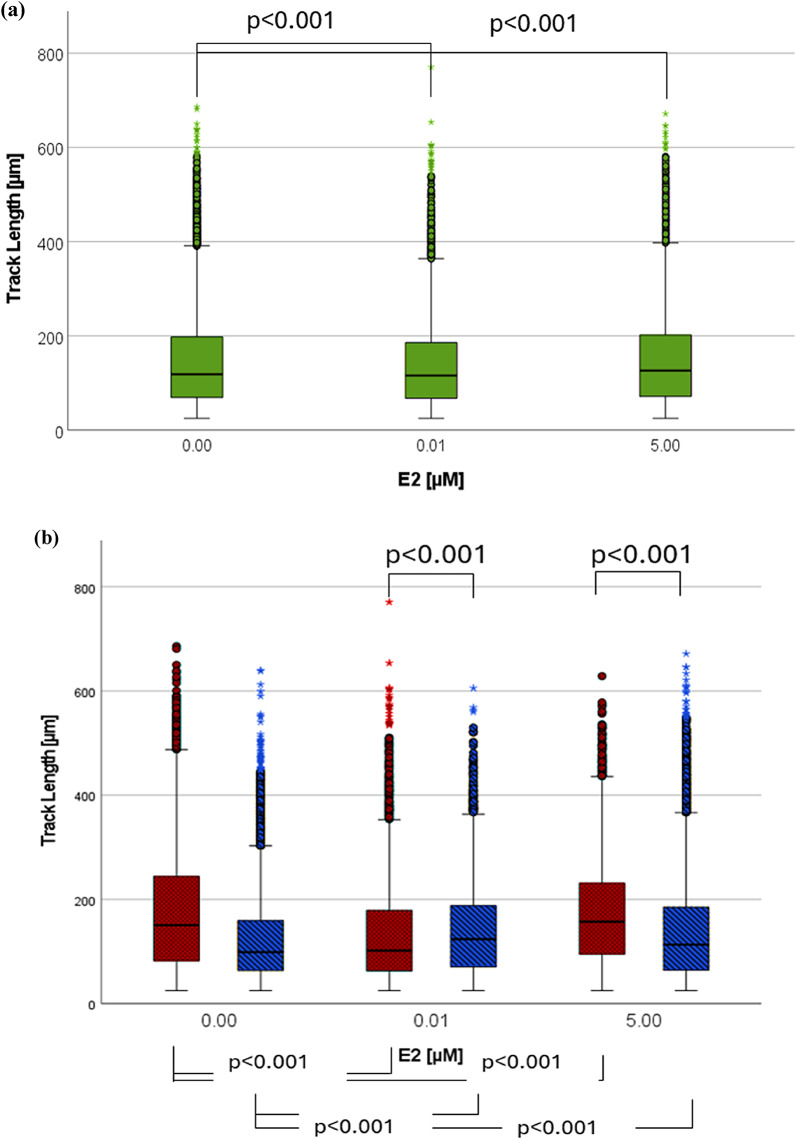
The impact of E2 on TL. (**a**) Analysis of six experiments with a balanced sex ratio, approximate track count of each box: 14.000-28.000. (**b**) Sex-specific results (female participants are represented by red dotted bars and male participants by blue diagonally striped bars). Approximate track count of each box: 5.000 – 15.000. Markings: (•) statistical outlier, (*) extreme statistical outlier, all non-centrifuged cells

TL with β-Estradiol [0.01 µM] (median: 116 μm, IQR: 118 μm) was significantly shorter as the control without E2 (zero sample) (median: 119 μm, IQR: 129 μm). High concentration of E2 [5 µM] led to a significant TL increase (median: 126 μm, IQR: 130 μm) (see Fig. 1a).

Among female participants (Fig. 1b), TL with β-Estradiol [0.01 µM] (median: 102 μm, IQR: 116 μm) was significantly lower in comparison to the control (median: 150 μm, IQR: 162 μm). TL with β-Estradiol [5 µM] was significantly higher (median: 157 μm, IQR: 137 μm). The TL of all zero samples from males was considerably short (median: 99 μm, IQR: 96 μm). In comparison, TL with β-Estradiol [0.01 µM] (median: 124 μm, IQR: 118 μm) as well as β-Estradiol [5 µM] (median: 113 μm, IQR: 121 μm) were significantly higher.

In an additional statistical analysis in which TL was treated as a technical replicate, the overall trends in TL changes remained consistent, with a significant effect observed for β-Estradiol [5 µM] (see Supplementary Fig. 3).

#### The impact of BPA on non-centrifuged cells

**Fig. 2:**
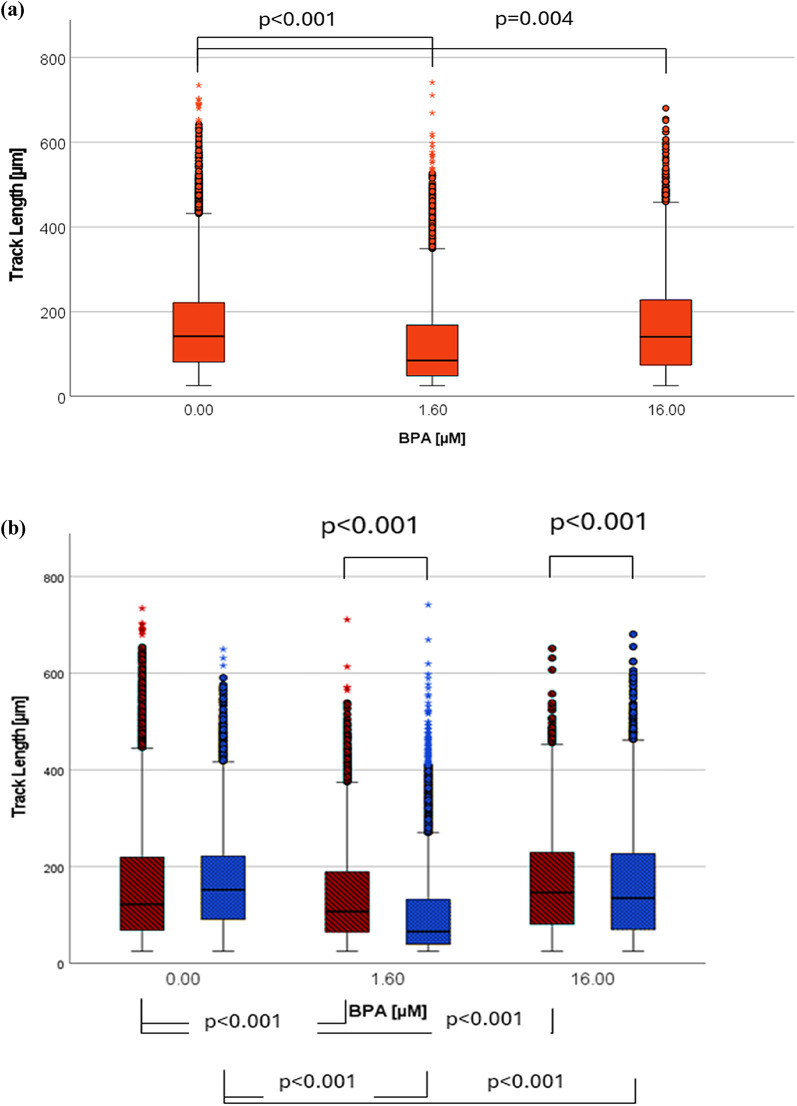
The impact of BPA on TL. (**a**) Analysis of six experiments with a balanced sex ratio, approximate track count of each box: 10.000-15.000. (**b**) Sex-specific results (female participants are represented by red dotted bars and male participants by blue diagonally striped bars). Approximate track count of each box: 5.000-9.000. Markings: (•) statistical outlier, (*) extreme statistical outlier, all non-centrifuged cells

Both concentrations of BPA led to a significant TL decrease (BPA [1.6 µM]: median: 85 μm, IQR: 120 μm, BPA [16 µM]: median: 140 μm, IQR: 154 μm, control: median: 142 μm, IQR: 140 μm) (see Fig. 2a). In a sex-specific approach (Fig. 2b), all results for BPA [1.6 µM] were significantly reduced (f/BPA [1.6 µM]: median: 107 μm, IQR: 125 μm, m/BPA [1.6 µM]: median 66 μm, IQR: 93 μm) in comparison to the control (f/zero sample: median: 122 μm, IQR: 151 μm, m/zero sample: median: 152 μm, IQR: 131 μm). Reduced TL was also seen regarding BPA [16 µM] and all male samples (median: 135 μm, IQR: 157 μm). A significant higher TL was shown among all women (median: 147 μm, IQR: 149 μm).

When TL was additionally analyzed as a technical replicate, the same patterns of change were observed, with statistical significance for BPA [1.6 µM] (see Supplementary Fig. 4).

#### The impact of E2 and BPA after centrifugation

TL of zero sample (centrifuged) was significantly lower in comparison to the control without centrifugation (zero sample: median: 155 μm, IQR: 151 μm, zero sample (centrifuged): median: 125 μm, IQR: 140 μm). Reduced TL was seen for all sexes (data not shown).

After centrifugation, E2 [0.01 µM] led to a significant TL decrease (median: 102 μm, IQR: 103 μm) when comparing to the TL of zero sample (centrifuged) (median: 128 μm, IQR: 147 μm). β-Estradiol [5 µM] (centrifuged) significantly diminished TL (median: 119 μm, IQR: 121 μm) (see Table 2). In an additional sex perspective, the same directions of change were seen among all samples of women (median: β-Estradiol [0.01µM] (centrifuged): 105 μm, IQR: 102 μm, β-Estradiol [5 µM] (centrifuged): median: 107 μm, IQR: 115 μm, zero sample (centrifuged): median: 136 μm, IQR: 156 μm). For all men there was a significant TL decrease when considering β-Estradiol [0.01 µM] (centrifuged) (median: 97 μm, IQR: 105 μm, zero sample (centrifuged): median 122 μm, IQR: 138 μm) (see Table 2).

TL of BPA [1.6 µM] (centrifuged) (median: 81 μm, IQR: 88 μm) was reduced when comparing to zero sample (centrifuged) (median: 120 μm, IQR: 132 μm). Within BPA [16 µM] (centrifuged) (median: 127 μm, IQR: 132 μm) there was a significant TL increase (see Table 2). Sex specific results were as follows: f/BPA [1.6 µM] (centrifuged): median: 75 μm, IQR: 69 μm, f/zero sample (centrifuged): median: 125 μm, IQR: 141 μm, m/ BPA [1.6 µM] (centrifuged): median: 88 μm, IQR: 102 μm, m/zero sample (centrifuged): median: 117 μm, IQR: 125 μm. A significant TL increase caused by BPA [16 µM] (centrifuged) was only seen among all men (median: 129 μm, IQR: 144 μm) (see Table 2).

**Table 2:** The impact of E2 and BPA on TL after centrifugation. Showing median TL, IQR and number of tracks. E2: Analyzing 11 experiments (f =female, *n*=5 and m =male, *n*=6). BPA: Analyzing 12 experiments (f =female, *n*=6 and m =male, *n*=6). Sex-specific data in the second and third column

	TL	IQR	Number of tracks	TL (f)	IQR	Number of tracks	TL (m)	IQR	Number of tracks
Zero sample (centrifuged)	128	147	23.696	136	156	10.773	122	138	12.923
β-Estradiol [0.01 µM] (centrifuged)	102	103	16.901	105	102	10.931	97	105	5.970
β-Estradiol [5 µM] (centrifuged)	119	121	13.072	107	115	5.807	129	123	7.265
Zero sample (centrifuged)	120	132	19.331	125	141	9.436	117	125	9.895
BPA [1.6 µM] (centrifuged)	81	88	10.559	75	69	4.977	88	102	5.582
BPA [16 µM] (centrifuged)	127	132	21.909	125	121	11.709	129	144	10.200

### Cell surface antigen expression

#### The impact of E2 on non-centrifuged cells

**Fig. 3:**
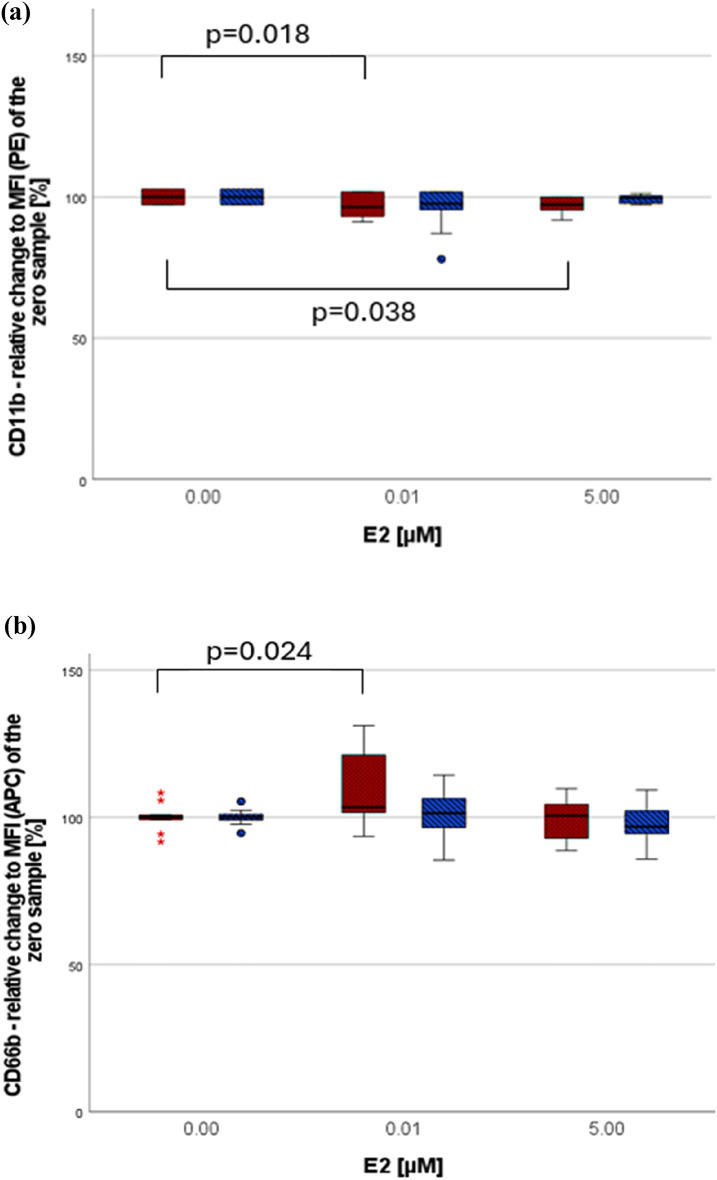
(**a**) The effect of E2 on CD11b. (**b**) The effect of E2 on CD66b. Data are presented as relative change [%] compared to the MFI of the zero sample (= no substance added). Marking: female participants = red dotted bars, male participants = blue diagonally striped bars, *n*=6 per sex, (•) statistical outlier, (*) extreme statistical outlier, all non-centrifuged cells

A significant decrease in the antigen expression of CD11b was evident at both tested E2 concentrations (Fig. 3a). E2 [0.01µM] led to a significant higher expression of CD66b (see Fig. 3b). This impact was limited to women.

#### The impact of BPA on non-centrifuged cells

**Fig. 4:**
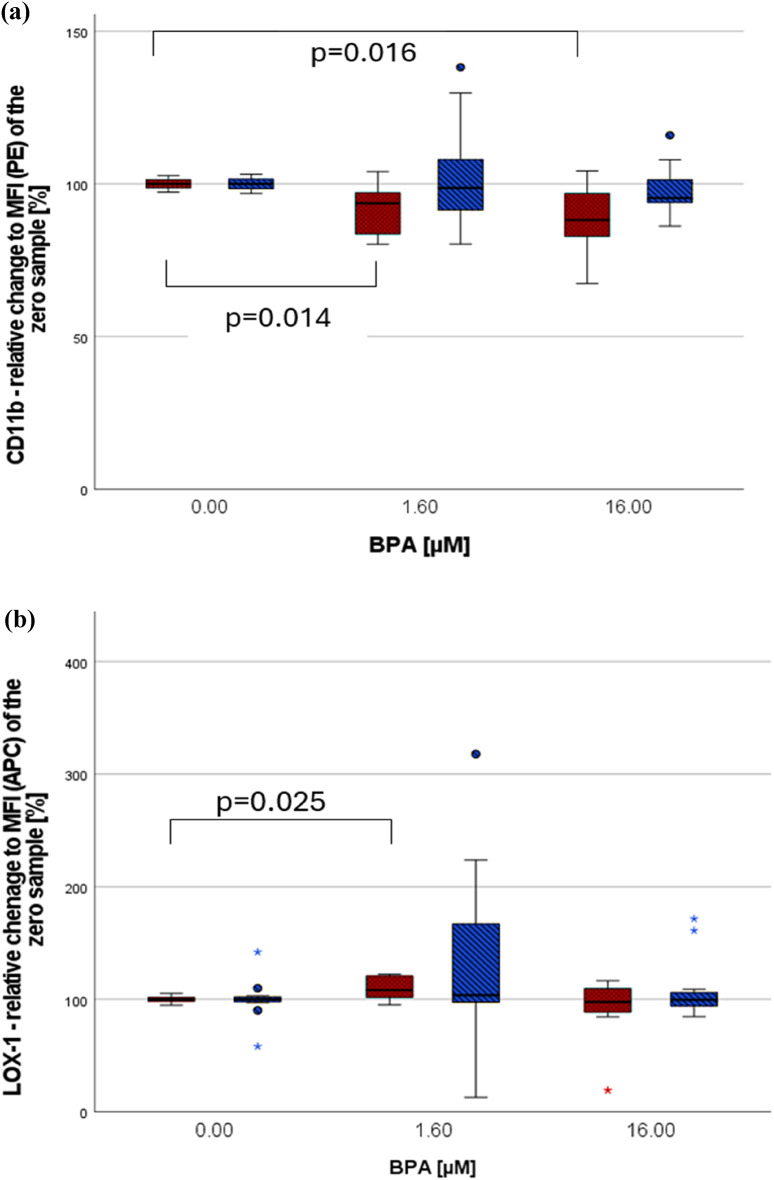
(**a**) The effect of BPA on CD11b. (**b**) The effect of BPA on LOX-1. Data are presented as relative change [%] compared to the MFI of the zero sample (= no substance added). Marking: female participants = red dotted bars, male participants = blue diagonally striped bars, *n*=6 per sex, (•) statistical outlier, (*) extreme statistical outlier, all non-centrifuged cells

Both concentrations of BPA significantly diminished the expression of CD11b on PMNs (see Fig. 4a). BPA [1.6 µM] caused a significant higher expression of LOX-1 (Fig. 4b). These effects were only seen among women.

#### The impact of E2 and BPA after centrifugation

After centrifugation, antigen expression of CD11b, CD66b and LOX-1 were at a significantly lower level as the control without centrifugation. Expression of CD62L was significantly higher (data not shown).

Both E2 concentrations significantly diminished CD11b expression on PMNs (*p* < 0.001). Among female samples and β-Estradiol [0.01 µM] (centrifuged), epitope density of CD62L appeared significantly lower (*p* < 0.001) (see Table 3).

Both concentrations of BPA led to a significant lower CD11b expression (*p* < 0.001). A significant increase in LOX-1 expression after both doses of BPA only showed within all male samples (*p* = 0.011 (BPA [1.6 µM] (centrifuged), *p* = 0.026 (BPA [16 µM] (centrifuged)). A higher CD62L epitope density was only seen among female participants and BPA [16 µM] (centrifuged) samples (*p* = 0.014) (see Table 3).

**Table 3:** The effect of E2 and BPA on antigen expression after centrifugation, indicated as relative changes to the median fluorescence intensity (MFI) of the zero sample (centrifuged) (= no substance added) ± standard deviation, (*n* = 12 with balanced sex-ratio for each substance). Significant results are printed in bold

	CD11b	CD66b	CD62L	LOX-1		CD11b	CD66b	CD62L	LOX-1
Zero sample (centrifuged)	100.00 ± 1.87	100.00 ± 2.30	100.00 ± 4.06	100.00 ± 7.67	Zero sample (centrifuged)	100.00 ± 1.47	100.00 ± 3.06	100.00 ± 3.64	100.00 ± 5.72
β-Estradiol [0.01 µM] (centrifuged)	**93.25 ± 6.28**	99.37 ± 8.41	91.32 ± 13.62		BPA [1.6 µM] (centrifuged)	**94.33 ± 6.72**	99.17 ± 10.26	101.01 ± 23.87	105.85 ± 12.10
β-Estradiol [5 µM] (centrifuged)	**95.63 ± 5.25**	99.37 ± 8.38	98.54 ± 10.80	101.66 ± 7.90		**92.21 ± 5.90**	95.95 ± 5.96	104.07 ± 19.04	106.66 ± 12.22
**Female**									
Zero sample (centrifuged)	100.00 ± 1.64	100.00 ± 2.16	100.00 ± 2.71	100.00 ± 10.25	Zero sample (centrifuged)	100.00 ± 1.69	100.00 ± 3.99	100.00 ± 4.37	100.00 ± 2.97
β-Estradiol [0.01 µM] (centrifuged)	**93.25 ± 3.03**	101.30 ± 5.78	**89.95 ± 8.76**	101.47 ± 10.49	BPA [1.6 µM] (centrifuged)	**95.37 ± 5.26**	100.00 ± 4.90	103.18 ± 30.05	102.81 ± 7.41
β-Estradiol [5 µM] (centrifuged)	**95.63 ± 6.13**	104.90 ± 6.11	96.14 ± 9.00	101.34 ± 4.88	BPA [16 µM] (centrifuged)	**92.21 ± 7.75**	95.95 ± 5.28	**112.90** ± **22.96**	106.66 ± 11.87
**Male**									
Zero sample (centrifuged)	100.00 ± 2.16	100.00 ± 2.53	100.00 ± 5.20	100.00 ± 4.24	Zero sample (centrifuged)	100.00 ± 1.30	100.00 ± 1.91	100.00 ± 2.94	100.00 ± 7.72
β-Estradiol [0.01 µM] (centrifuged)	**94.35 ± 8.34**	96.69 ± 9.93	97.82 ± 13.93	106.07 ± 68.60	BPA [1.6 µM] (centrifuged)	**92.64 ± 7.88**	98.52 ± 13.99	96.56 ± 12.42	**106.51** ± 14.78
β-Estradiol [5 µM] (centrifuged)	**95.62 ± 3.86**	93.98 ± 6.92	100.80 ± 12.56	104.37 ± 10.31	BPA [16 µM] (centrifuged)	**93.05 ± 2.83**	96.02 ± 6.74	98.50 ± 5.37	**107.91** ± 13.02

### Oxidative burst

#### The impact of E2 on non-centrifuged cells

**Fig. 5:**
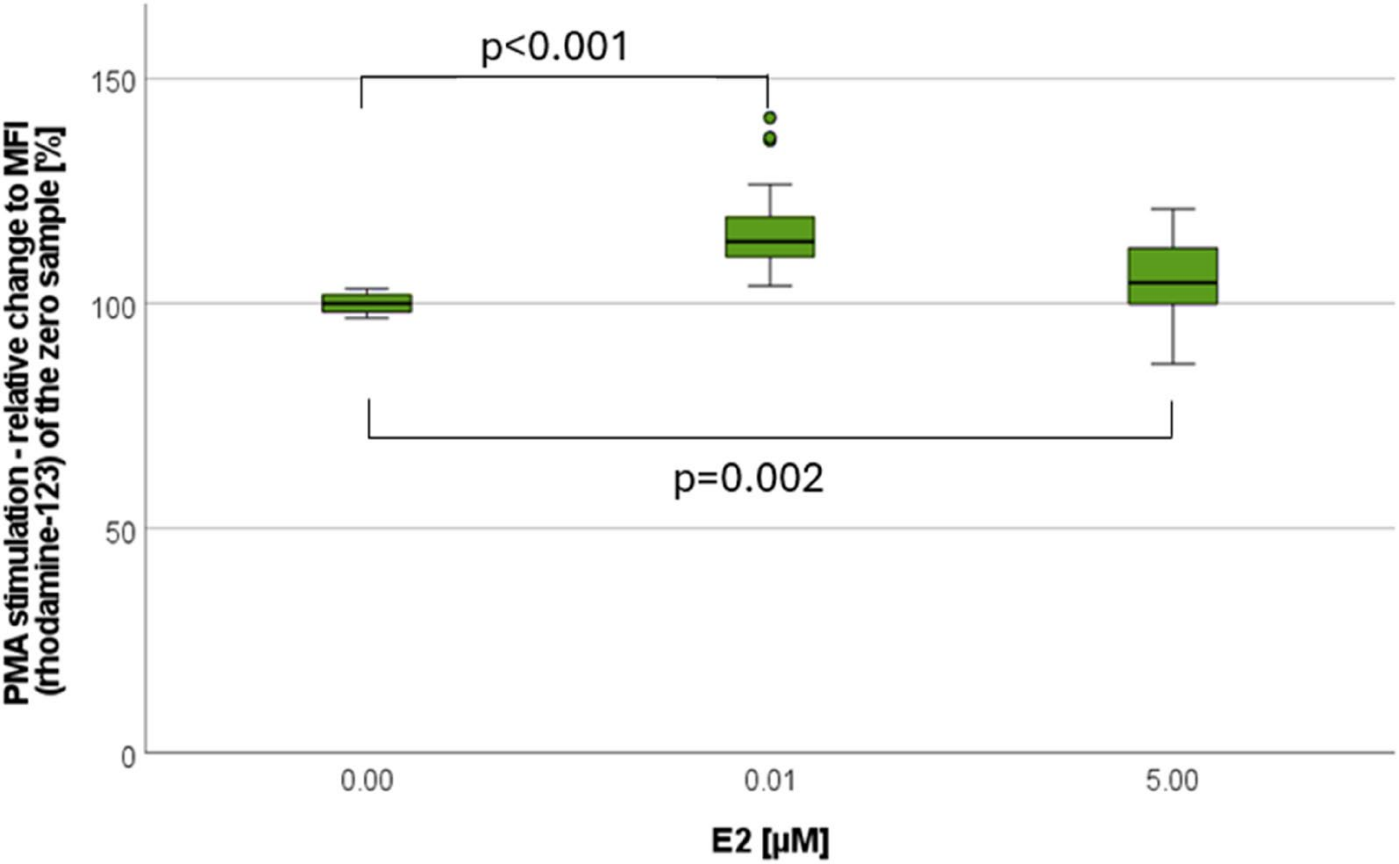
Burst - E2-effect under PMA stimulation. Analyzing 12 experiments with balanced sex-ratio. Presentation as relative change [%] in relation to the MFI of the zero sample (= no substance added). Marking: (•) statistical outlier, all non-centrifuged cells

After PMA stimulation, the low dose of E2 [0.01 µM] led to a significant higher change as the high dose of 5 µM, when comparing to the control (see Fig. 5). In all samples of men this significant effect was only seen within β-Estradiol [0.01 µM]. No significant results were observed following fMLP stimulation.

#### The impact of BPA on non-centrifuged cells

**Fig. 6:**
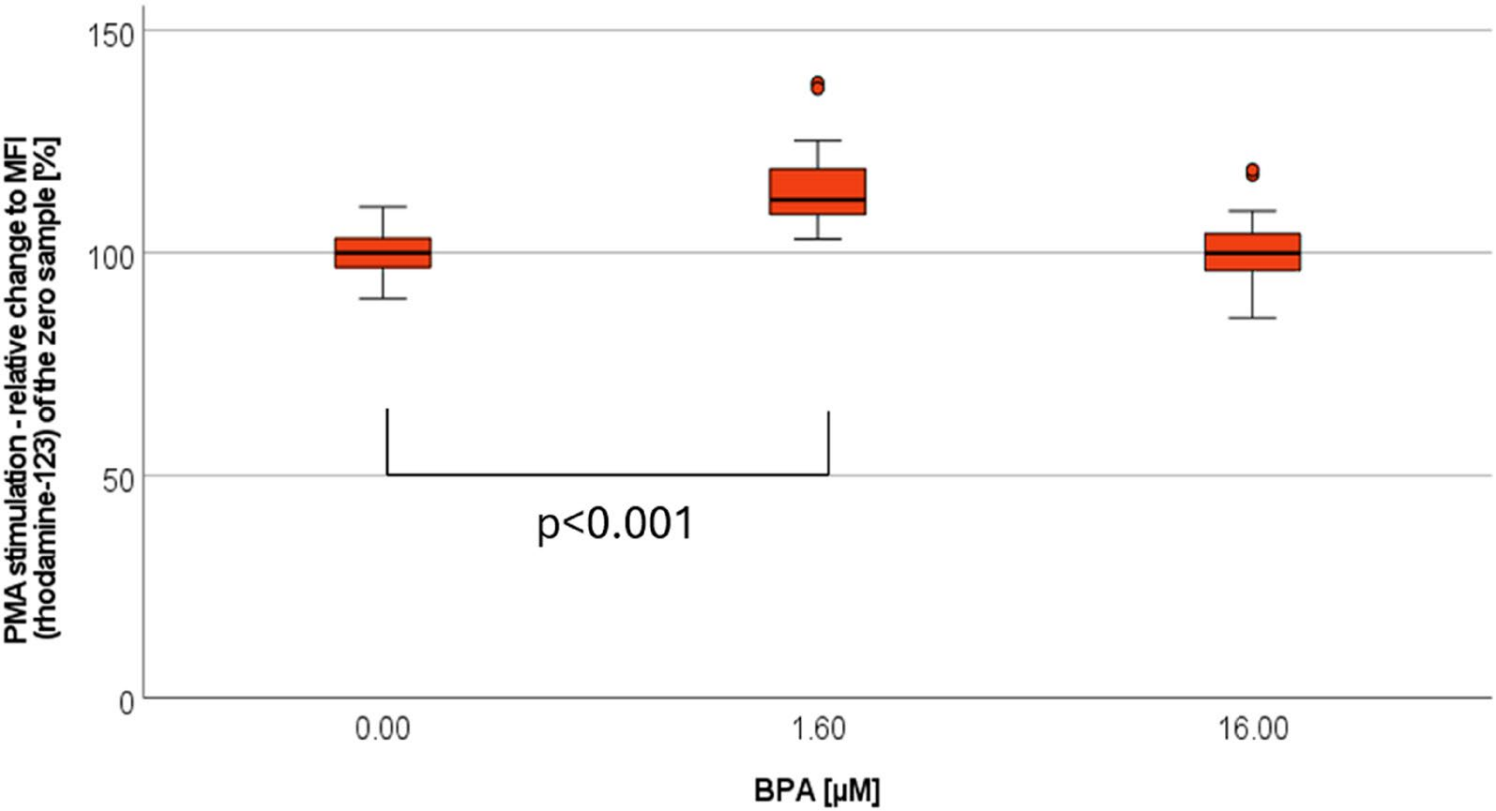
Burst - BPA-effect under PMA stimulation. Analyzing 12 experiments with balanced sex-ratio. Presentation as relative change [%] in relation to the MFI of the zero sample (= no substance added). Marking: (•) statistical outlier, all non-centrifuged cells

In a sex-specific analysis of BPA [1.6 µM], significant changes were only seen after PMA stimulation, when comparing to the control (see Fig. 6).

#### The impact of E2 and BPA after centrifugation

PMA and fMLP samples showed a significant difference when comparing centrifuged cells with non-centrifuged samples (data not shown). After PMA stimulation there was a significant difference between zero sample (centrifuged) and β-Estradiol [0.01 µM] (centrifuged) (see Table 4). This applied to all sexes. fMLP-samples of women showed a significant difference between zero sample (centrifuged) and BPA [16 µM] (centrifuged). PMA-activation led to a significant difference between zero sample (centrifuged) and BPA [1.6 µM] (centrifuged). There were no significant results among all samples of men (see Table 4).

**Table 4:** The effect of E2 and BPA on oxidative burst after centrifugation, indicated as relative changes to the median fluorescence intensity (MFI) of the zero sample (centrifuged) (= no substance added) ± standard deviation, (*n* = 12 with balanced sex-ratio for each substance). Significant results are printed in bold

	PMA	fMLP		PMA	fMLP
Zero sample (centrifuged)	100.00 ± 2.36	100.00 ± 6.13	Zero sample (centrifuged)	100.00 ± 2.38	100.00 ± 13.72
β-Estradiol [0.01 µM] (centrifuged)	**106.76 ± 6.42**	101.53 ± 7.83	BPA [1.6 µM] (centrifuged)	108.64 ± 6.45	113.15 ± 31.47
β-Estradiol [5 µM] (centrifuged)	102.22 ± 3.87	100.17 ± 13.12	BPA [16 µM] (centrifuged)	96.85 ± 8.42	109.28 ± 32.48
**Female**					
Zero sample (centrifuged)	100.00 ± 2.28	100.00 ± 6.92	Zero sample (centrifuged)	100.00 ± 3.10	100.00 ± 3.92
β-Estradiol [0.01 µM] (centrifuged)	**106.93 ± 5.58**	100.00 ± 6.82	BPA [1.6 µM] (centrifuged)	**109.86 ± 3.05**	101.83 ± 22.67
β-Estradiol [5 µM] (centrifuged)	101.58 ± 4.41	90.80 ± 16.39	BPA [16 µM] (centrifuged)	98.62 ± 5.47	**121.72 ± 17.52**
**Male**					
Zero sample (centrifuged)	100.00 ± 2.53	100.00 ± 5.54	Zero sample (centrifuged)	100.00 ± 1.48	100.00 ± 19.45
β-Estradiol [0.01 µM] (centrifuged)	**106.61 ± 6.99**	103.26 ± 8.77	BPA [1.6 µM] (centrifuged)	105.54 ± 8.28	125.70 ± 35.06
β-Estradiol [5 µM] (centrifuged)	102.85 ± 3.10	103.08 ± 8.10	BPA [16 µM] (centrifuged)	95.60 ± 10.87	106.45 ± 43.26

## Discussion

### Migration

**E2 and BPA influence chemotactic migration.**

#### E2:

In all experiments with the substance E2, the effects of two different active concentrations were assessed. While previous studies have largely investigated lower, physiological concentrations, this study explores higher doses to provide further insights and broaden the understanding of the field [11, 12, 14]. The physiological concentration of this sex hormone in the human body averages 0.1 nM [14]. With β-Estradiol [0.01 µM] a corresponding dilution in 100-fold concentration was used. In order to examine a supramaximal response, our selection of β-Estradiol [5 µM] was guided by the rationale proposed by Yasuda et al. [13]. It is conceivable that, within specific tissues such as the placenta, locally elevated estrogenic microenvironments may exist that exceed circulating concentrations [13, 14, 20]. In the corresponding test series, β-Estradiol [0.01 µM] led to a significant shorter TL. In comparable studies, the effect of different concentrations of E2 (0.1 nM − 1000 nM) on fMLP-mediated migration was investigated (see Supplementary Table 4) [11, 12, 14]. Ratajczak-Wrona et al. [14] described no change in chemotaxis at 0.1 nM compared to the control. In contrast, an inhibitory effect was observed by Zhang et al. [11] with only a slight increase in the hormone dose (1 nM). This influence was also present at other concentrations (0.1 nM -1000 nM) with a dose-dependent effect relationship [12]. Our results for β-Estradiol [0.01 µM] are consistent with those reported by Zhang et al. [11] and Ito et al. [12]. When analyzed individually by sex, a TL increase was seen among men. Whether the reason for that was the low TL of the zero samples requires further investigation. Ratajczak-Wrona et al. [14] also took a differentiated approach depending on sex. They found no sex-specific difference. The study of Zhang et al. [11] explored the effects of E2 at concentrations of 1 nM and 100 nM exclusively in male subjects. Ito et al. [12] initially demonstrated a significant dose-dependent chemotactic migration impairment (0.1–1000 nM E2) in men with endogenous plasma levels of estrogen less than 0.5 nM. In addition, physiological concentrations of E2 during the menstrual cycle (0.1-1.0 nM) inhibited female PMN chemotaxis in vitro, with higher concentrations correlating with a greater reduction in chemotactic activity, reflecting an inverse relationship. A sex independent TL increase was seen when investigating β-Estradiol [5 µM]. To the best of our knowledge, the effect of such high E2 hormone dosage on chemotactic migration has not been analyzed yet. Therefore, this result supplies new insights in migration of PMNs. For further findings, a study analogous to the procedure of Ito et al. [12] appears to be useful.

#### BPA:

The study also evaluated the effect of two different concentrations of BPA. Due to frequent, partly unconscious everyday exposure to this EDC, an average BPA concentration of approximately 16 nM has been reported in human serum [14]. In order to broaden the scope of research and uncover possible inverse or counterintuitive effects, we employed a dose 100 times (1.6 µM) and 1000 times (16 µM) greater. Hereby BPA [1.6 µM] led to a significant decrease in TL. This outcome was observed regardless of sex. Reference literature showed a similar effect [14, 15] (see Supplementary Table 4). Balistrieri et al. [15] described a significant impairment of fMLP-mediated chemotaxis by PMNs after incubation with BPA at concentrations of 0.1 and 1 µM. In addition, a reduction in chemotaxis was observed at 16 nM and 1.6 µM BPA, independent of donor sex [14]. Supraphysiological concentration of this EDC present in the body thus appears to have a negative effect on the migration behavior of PMNs. One comparable study analyzed the relative elimination of methicillin-resistant Staphylococcus aureus (MRSA) by PMNs after incubation with BPA at concentrations of 10–100 µM. Notably, only the highest concentration caused a significant increase in MRSA survival [15]. In our test series there was only a decrease in TL after cell incubation with BPA [16 µM] in all samples of men. Similar to the β-Estradiol [5 µM]-test series the TL increased when analyzing samples of women. This could probably show a nonmonotonic dose-effect relationship (NMDR), which is described in the context of E2 and some EDCs [14, 21]. This phenomenon is, among other aspects, attributed to the very complex mode of action of these substances [10, 21, 22]. Several studies have demonstrated that BPA and related compounds influence cellular functions via a variety of receptors and signaling cascades [23]. Notably, both, genomic and rapid non-genomic signaling pathways are described. An overview, including specific reference to the effect of BPA and E2 on PMNs, is summarized in the Supplementary (see Supplementary Table 3). Sex-specific differences may be attributable to variations in affinity and concentration, as well as competition with endogenous hormones [14, 21, 24]. Women have significantly higher levels of endogenous E2, which may lead to partially saturated estrogen receptor (ER) signaling pathways. Consequently, male PMNs may exhibit differential sensitivity to xenoestrogenic stimulation by BPA [14]. Further investigation of the precise cellular mechanisms by which BPA affects PMNs, as well as potential sex-specific differences in signaling pathways and metabolic programming, could provide greater clarity in this context [25].

**Centrifuged PMNs migrate significantly shorter.** A previous study of Hundhammer et al. [19] demonstrated that PMNs are affected in their functionality in a 'g-time' dependent manner. The newly introduced parameter 'g-time' is defined by the two variables g-force (RCF) [g] and centrifugation time (CT) [s]. g-time [*g*s] = (RCF [*g*] × CT [s]) [19].

Our studies were able to confirm the inhibitory effect of centrifugation on chemotactic migration as it has been investigated [19]. After the preparation of a PMN-rich supernatant using Gelafundin, our samples were centrifuged with a 'g-time' of 90 k*g*s (300*g* for 300 s). Hereby, a sex-independent TL-decrease was seen when comparing with non-centrifuged cells. In comparison to that, the reference project studied the influence of centrifugation in two different ways [19]. On the one hand, they analyzed the impact of four different RCFs (10*g*, 20*g*, 30*g*, 47*g*) during constant centrifugation duration of 10 min. On the other hand, different CTs of 5 and 15 min at 20*g* and 30*g* were compared with each other. As a result, both aspects led to a significant deviation of the TL when comparing to the control. When analyzing the parameters RCF and CT together as 'g-time', lowest TL-values have been seen above values over 30 k*g*s. Our results enabled us to extend this study by obtaining similar results at a 'g-time' of 90 k*g*s.

**E2 and BPA impair chemotactic migration regardless of centrifugation.** The impact of centrifugation, as observed in our experiments, leads to the question whether and to what extent the substances E2 and BPA exert influence on centrifuged PMNs. Are the cells already impaired to such an extent, so that the EDC and hormone do not cause any further impairment? Is there an additive or contradictory effect? This aspect is also interesting against the background of research work that has already been carried out and includes centrifugation either for isolation or in another work step [8, 11, 12, 14, 15, 26]. Do they genuinely reflect hormonal effects, or might they be systematically biased by centrifugation?

After centrifugation, both concentrations of E2 led to a significant TL decrease. When comparing these results with the first test series of our project (no centrifugation) we would have only expected this effect for all β-Estradiol [0.01] (centrifuged) samples. Contrasting results were seen in the β-Estradiol [5 µM] (centrifuged) test series. Further investigations led us to a sex-specific consideration. Hereby we saw noticeable differences between samples of men and women. All male samples showed similar results to the first test series, whereby β-Estradiol [5 µM] (centrifuged) results did not reach a significant level.

The TL of centrifuged cells was significantly impaired by BPA. Analogue to the first test series, BPA [1.6 µM] (centrifuged) led to a sex-independent significant TL decrease. In contrast to that, BPA [16 µM] (centrifuged) showed a significant increase among all men.

In summary, both the hormone and the EDC seem to impact chemotactic migration of non-centrifuged and centrifuged PMNs. Centrifugation alone at a 'g-time' of 90 k*g*s does not impair PMNs to such an extent, so that the analyzed substances could no longer exert any effect. In which way different concentrations exert which directional effect on the TL of the PMNs and the role of sex aspects needs to be investigated in further research.

### Cell surface antigens

**E2 and BPA influence cell surface expression.** PMNs express a range of different antigens on their cell surface. The so called cluster of differentiation (CD) are building the genetic determined immunophenotype and fulfill various tasks [2]. In our studies, we analyzed the expression of CD11b, CD66b, CD62L and LOX-1. First, after cell incubation with E2 or BPA, both concentrations of these substances led to a relative decrease of CD11b. This best-known integrin receptor on PMNs (Mac-1, αMβ2) forms a heterodimer together with CD18 and is composed of an α- and β-subunit. More than 40 different ligands can bind to this complex. This surface molecule is involved in the activation of different granulocyte functions and, to a certain extent, represents the status of cell activity [27]. From a clinical perspective, the reduced CD11b expression could compromise host defense, leading to increased susceptibility to infections, particularly in vulnerable populations.

A significant increase of CD66b was caused solely by β-Estradiol [0.01 µM]. As one of four 'carcinoembryonic antigens', CD66b (CECAM8) belongs to the family of cell adhesion molecules [3]. In considering possible clinical relevance, CD66b PMNs have been associated with enhanced NET formation and potential immunothrombotic activity in stroke patients [28]. The involvement of E2 in this process of overactivation and related vascular complications remains unclear and would require further investigation.

Only BPA [1.6 µM] caused a significant increase in LOX-1. This scavenger receptor is expressed on a variety of cell types and was originally identified as a receptor mediating the uptake of oxidized low-density lipoprotein (oxLDL) [29]. LOX-1 is in the center of interest in many studies about cardiovascular diseases [30]. Due to the increased expression of LOX-1 by BPA, this EDC could be discussed as a possible influencing factor in the formation of atherosclerotic plaques and cardiovascular diseases. In a broader context, various studies have shown that the proportion of LOX-1 on PMNs is elevated under different pathological conditions (for example allergy or severe infection) [31–33]. The potential role of BPA as an individual contributor to this presumably multifactorial phenomenon and its amplifying effect remains to be elucidated.

All these significant effects were observed exclusively in samples of females. Therefore, the impact on cell surface expression seemed to be sex-specific in our studies.

In comparison with other studies (see Supplementary Table 5), physiological doses of E2 did not affect the expression of CD11b, CD33 or CD34 [8]. Similarly, the expression of CD14 and CD284 remained unchanged [14]. In addition, the effects of BPA on selected antigens were examined by Ratajczak-Wrona et al. [26]. In contrast to our studies their results showed almost sex-independent, whereby female PMNs seemed to be more affected. In female test subjects, exposure to BPA at different concentrations (16 nM, 1.5 µM, 3 µM, 6 µM and 12 µM) resulted in a reduced percentage expression of CD11c, CD15 and CD16. Next to that, an increased CD62L expression was observed after incubation with the highest BPA concentration (12 µM), an effect that was not detected in our study. Moreover, only exposure to 16 nM BPA led to an increased CD14 ratio [26]. Comparable effects were reported in male test subjects, with the exception of CD11c, which showed a decrease only after incubation with 12 µM BPA. No changes in CD62L expression were found in men [26].

In the course of exploring possible sex-specific differences, data show that estrogen and androgen signals as well as genetic factors modulate neutrophil maturation, function and activity [34]. PMNs from healthy adult women have a pre-activated phenotype, which is characterized by increased expression of type I interferon-stimulated genes [35]. Overall, women are reported to have stronger antimicrobial defenses, but also a higher risk of autoimmune and inflammatory diseases [34]. Thus, one possible explanation for the relative gender-specific changes in antigen expression could be that substance effects only become significant at specific doses and in female cells with fundamentally stronger activity.

**Centrifugation influences cell surface expression.** The influence of centrifugation on cell surface expression can again be compared with the studies of Hundhammer et al. [19]. As a different methodological approach, we measured relative change in antigen expression after 3-hour incubation of the centrifuged cells at 37 °C. By contrast, the comparative study assessed median fluorescence intensities (MFIs) immediately after isolation and following a 22-hour incubation with the stimulator fMLP (t = 0; t = 22).

After treating PMNs with a 'g-time' of 90 k*g*s, we saw a relative decrease in CD11b and CD66b expression. Hundhammer et al. [19] did not detect significant changes in CD11b or CD66b expression at t = 0. Following incubation with fMLP and subsequent analysis, CD11b expression was significantly reduced when analyzing 'g-times' of 42 k*g*s and 680 k*g*s. In contrast, PMNs isolated with 1 or 9 k*g*s, showed a relative increase in CD11b expression. Similar patterns were observed for CD66b, with an inhibitory effect evident at 680 k*g*s. Certain 'g-times' thus seem to decrease CD11b and CD66b expression, affecting both their basal levels and their capacity to respond to fMLP stimulation. CD62L, analyzed as another antigen, behaves differently from CD11b and CD66b, as its expression decreases upon increased cell activation [36]. A PMN impairment can be derived from the significant increase of this marker after treatment with 90 k*g*s and 3-hour incubation. In contrast to that, studies of Hundhammer et al. [19] showed a reduction of CD62L. This result appeared independent of 'g-time' and 22-hour incubation with fMLP. As an extension, we have also analyzed the surface marker LOX-1. After 90 k*g*s, a significant decrease was observed. Overall, centrifugation influences cell surface antigen expression of PMNs. This result shows that a careful choice of the isolation method is necessary.

**E2 and BPA impair cell surface expression regardless of centrifugation.** Incubation of centrifuged PMNs with both concentrations of E2 and BPA resulted in a decrease in CD11b expression. Therefore, comparable impacts on this cell surface antigen were detected following the individual application of the two substances, centrifugation, and when both effects were considered together. In contrast to our first study line, there were no sex-specific differences. Analysis of BPA and LOX-1 yielded results similar to those of the first test series. Non-centrifuged, as well as centrifuged cells showed an increase in this cell surface antigen after incubation with 1.6 µM BPA. Moreover, this effect was also seen for BPA [16 µM] (centrifuged) samples, but not for BPA [16 µM]. The sex effect appeared inconsistent. We did not see any standardized patterns in CD62L and CD66b expression, when including the centrifugation analysis. As a conclusion, despite 90 k*g*s-centrifugation, both substances led to an influence in cell surface expression. In which extent different 'g-times' could cause a possible overlay effect, warrants further investigation.

### Oxidative burst

**E2 and BPA influence ROS release after PMA-stimulation.** The influence of E2 on ROS release has been extensively discussed in the literature. The results vary depending on the methodology used in the different study protocols (see Supplementary Table 6). Next to that, sex seems to play a specific role [8]. When using the intracellular dye DHR123 and PMA-stimulation, female cells showed an increased ROS production. Hereby, PMNs were incubated with physiological doses (10^− 12^ – 10^− 7^ g/mL) for 6 h [8]. Additionally, also PMNs from pregnant women seemed to release more intracellular ROS-intermediates [37, 38]. Extracellular ROS production has been reported to decrease upon exposure to physiological doses of E2, with similar findings observed in studies involving pregnant women [8, 39–41]. Consequently, E2 is said to have an antioxidative effect [40, 42]. Our studies showed similar results regarding PMA-samples. Exposure to both E2 concentrations induced a pronounced oxidative burst in female test subjects. In samples from male participants, this effect was detectable only after β-Estradiol [0.01 µM] incubation. A possible explanation for this could be sex-specific differences in the expression of protein kinase C, which is activated by PMA [8, 43, 44]. A concentration, wide above physiological doses, seems not to cause a significant increase in men. There was no significant difference after fMLP-stimulation in our research. BPA also appears to exert an influence on ROS production. Different projects have shown a ROS-induction through BPA or the BPA-derivate Tetrabromobisphenol A (TBBPA) [14, 15, 45]. The mechanism underlying BPAʼs effect on ROS release is likely modulated by ERβ activation and the involvement of calcium signaling [15]. In our experiments we saw an increase in ROS-production after PMA-stimulation. This effect was limited to a concentration of 1.6 µM. The concentration-dependent ROS induction reported by Balistrieri et al. [15] for doses ranging from 0.03 to 100 µM was not reproduced in our study, owing to the lack of significant effects for BPA [16 µM].

**Centrifugation influences ROS release after fMLP and PMA-stimulation.** As previously, the analysis of centrifuged PMNs was conducted considering two distinct stimulation modalities. Exposure to the '*g*-time' of 90 k*g*s led to a relative change in ROS-production after PMA as well as after fMLP stimulation. Oxidative burst was observed at a significantly elevated level when comparing with non-centrifuged PMNs. Hundhammer et al. [19] found a negative correlation for all '*g*-times' (9, 18, 27, 42 and 680 k*g*s) after fMLP-stimulation when comparing with the control group (1*g*). The lowest 'g-time', reaching statistical significance, was between 27 and 42 k*g*s. These two samples also showed a ROS-decrease after PMA-stimulation. In contrast to that, there was an increase in oxidative Burst for 680 k*g*s (*p* < 0.05). These results implicate, that centrifugation influences both, cell surface mediated as well as intracellular Burst. Our analysis of an additional '*g*-time' (90 k*g*s) indicates that the magnitude of this centrifugation parameter appears to play a decisive role in determining the direction of its impact. The most negative effect was seen at 'g-times' of 27 and 42 k*g*s [19]. Exposure to a 'g-time' of 90 k*g*s, about twice the second-mentioned value, led to elevated ROS production, an effect that was likewise seen at 680 k*g*s. More findings on affected signaling pathways could help in further explanation.

**Low dose E2 and BPA impair ROS release regardless of centrifugation.** After PMA-stimulation, β-Estradiol [0.01 µM] (centrifuged) samples showed a sex-independent increase in oxidative burst, corresponding to the non-centrifugation setup. There was no effect after incubation with the high E2 dose of 5 µM, which has been shown before among all women and non-centrifuged cells. After centrifugation and addition of BPA, a significant impact was only seen in female BPA [1.6 µM] (centrifuged) samples. The significant result for BPA [16 µM] (centrifuged) in fMLP-stimulated female samples cannot be explained by us any further. All in all, the results of the first test series were only partially reproduced after centrifugation. Particularly noticeable is the missing increase in PMA-stimulated Burst and the higher substance concentrations, respectively. One possible explanation could be a nonmonotonic dose-effect relationship (NMDR). Assuming that maximum effect is caused by low concentration, centrifugation could influence PMNs in such an extent, that possibly already less effective high concentration cannot lead to a significant change any longer.

## Conclusion

Our study investigated whether the sex hormone 17-β-estradiol and the endocrine-disrupting chemical Bisphenol A exert an influence on neutrophil functions and surface epitopes.

E2 significantly influenced migration behavior, cell surface expression and oxidative burst of PMNs. The effect seemed to be sex-specific, with women being more affected. Against the background of naturally different hormone levels in women and men, this could provide a valuable insight into the relationship between the role of PMNs and sex-specific autoimmunity.

Our study of the endocrine-disrupting chemical BPA confirmed and expanded existing research work by an additional analysis of high substance concentration (16 µM). The significant impact of BPA on PMNs shows the importance of limited usage of this EDC in everyday life.

We were able to confirm the paralytic effect of centrifugation on neutrophil functions. Centrifugation at a 'g-time' of 90 k*g*s (300*g* for 300 s) does not impair PMNs to such an extent, so that the analyzed substances could no longer exert any effect. Nevertheless, we suggest the choice of a gentle isolation method, avoiding centrifugation. The impact of high 'g-times', as they have been used in different study protocols, could be substance of further research.

## Electronic Supplementary Material

Below is the link to the electronic supplementary material.

Supplementary Material 1

## Data Availability

The datasets used and analyzed during the current study are available from the corresponding author on reasonable request.

## Abbreviations

ANOVA: One-way analysis of variance
APC: Allophycocyanin
BPA: Bisphenol A
CD: Cluster of differentiation
CT: Centrifugation time
DHR: Dihydrorhodamine
E2: 17-β-estradiol
EDC: Endocrine disrupting chemical
ER: Estrogen receptor
f: Female
FITC: Fluorescein
fMLP: N-formyl-methionyl-leucyl-phenylalanine
IQR: Interquartile range
m: Male
MFI: Median fluorescence intensity
MRSA: Methicillin-resistant Staphylococcus aureus
NMDR: Nonmonotonic dose-effect relationship
oxLDL: Oxidized low-density lipoprotein
PBS: Dulbecco’s phosphate-buffered saline solution with/without MgCl_2_ and CaCl_2_
PE: Phycoerythrin
PI: Propidium Iodide
PMA: Phorbol 12-myristate 13-acetate
PMNs: Polymorphonuclear leukocytes
RCF: g-force
ROS: Reactive oxygen species
RPMI: Roswell Park Memorial Institute
SNARF: Seminaphtharhodafluorine
TBBPA: Tetrabromobisphenol A
TL: Track length
TNFα: Tumor necrosis factor α
