# Supplementary material for: Sex-specific effects of 17-β-estradiol and bisphenol A on neutrophil function and phenotype – does centrifugation matter?
Source: Biol Sex Differ. 2026 Apr 24;17:90. doi: 10.1186/s13293-026-00906-9 (PMC13123024; doi:10.1186/s13293-026-00906-9)
Supplement: Supplementary file 1 — Supplementary Material 1 [file 13293_2026_906_MOESM1_ESM.docx]

# Supplementary

Supplementary Table 1: Detailed participant data for the analysis of E2 and BPA, categorized by live-cell imaging and flow cytometry. All participants were healthy volunteers with no known relevant pre-existing conditions. Data are presented as median [min–max]. (a) Live-cell imaging: each concentration was analyzed with n = 6 participants (centrifuged and non-centrifuged test series), except for Estradiol 5 [µM] (centrifuged) (n = 5). Equal representation of sexes was maintained. (b) Flow cytometry: each concentration was analyzed with n = 12 participants (centrifuged and non-centrifuged test series). A balanced sex ratio was ensured.

**(a)**

| Live-cell imaging: substance analysis of E2 and BPA on non-centrifuged cells and centrifuged cells | | | | |
| --- | --- | --- | --- | --- |
| Substance | Sex [f, m] | Age [years] | Height [cm] | Weight [kg] |
| E2 | f | 24 [21,31] | 171.5 [163,180] | 60 [50,76] |
|  | m | 30 [21,58] | 179 [171,189] | 79 [68,85] |
| BPA | f | 24 [22,31] | 168 [163,175] | 63 [50,75] |
|  | m | 23 [21,31] | 179 [177,187] | 75 [62,88] |

**(b)**

| Flow cytometry: substance analysis of E2 and BPA on non-centrifuged cells and centrifuged cells | | | | |
| --- | --- | --- | --- | --- |
| Substance | Sex [f, m] | Age [years] | Height [cm] | Weight [kg] |
| E2 | f | 24 [21,57] | 168 [163,179] | 63 [50,79] |
|  | m | 30.5 [21,58] | 176 [171,183] | 78 [68,84] |
| BPA | f | 22.5 [21,30] | 168 [168,179] | 64.5 [54,75] |
|  | m | 27 [22,37] | 184 [179,187] | 73.5 [62,98] |

Supplementary Table 2: Sample classification (samples that have been spiked with BPA are assigned the color orange, E2-spiked samples are color-coded green). All samples that underwent centrifugation are marked accordingly, as indicated in parentheses (centrifuged).

| Sample name | Explanation |
| --- | --- |
| Zero sample | Untreated cells |
| Zero sample (centrifuged) | Centrifuged cells |
| β-Estradiol [5 µM] | Cells treated with E2 at a concentration of 5 µM |
| β-Estradiol [0.01 µM] | Cells treated with E2 at a concentration of 0.01 µM |
| β-Estradiol [5 µM] (centrifuged) | Centrifuged Cells treated with E2 at a concentration of 5 µM |
| β-Estradiol [0.01 µM] (centrifuged) | Centrifuged Cells treated with E2 at a concentration of 0.01 µM |
| BPA [16 µM] | Cells treated with BPA at a concentration of 16 µM |
| BPA [1.6 µM] | Cell treated with BPA at a concentration of 1.6 µM |
| BPA [16 µM] (centrifuged) | Centrifuged Cells treated with BPA at a concentration of 16 µM |
| BPA [1.6 µM] (centrifuged) | Centrifuged Cells treated with BPA at a concentration of 1.6 µM |

**(a)**

**(b)**

fMLP + autologous serum

Channel: collagen matrix

Chamber 1: Zero sample

Chamber 2 (one of the following samples, depending on the test series):

β-Estradiol [5 µM]

BPA [16 µM]

Zero sample (centrifuged)

Chamber 3 (one of the following samples, depending on the test series):

β-Estradiol [0.01]

BPA [1.6 µM]

β-Estradiol [5 µM] (centrifuged)

β-Estradiol [0.01 µM] (centrifuged)

BPA [16 µM] (centrifuged)

BPA [1.6 µM] (centrifuged)

Left reservoir

Right reservoir

Supplementary Figure 1: (a) Schematic illustration of one 3D-µ-Slide chamber. Each slide consists of three chambers with separate channels, which are bordered on both sides by two reservoirs. PMNs were introduced into the right reservoir and migrated through the channel to the attractant in the left reservoir. (b) Detailed description of the various suspensions added. While fMLP + autologous serum was always added to the left reservoir and a collagen matrix was always added to the channel, the filling of the right reservoir varied depending on the test series (see right column of table).

| **Left reservoir (65 µL)** | **Channel (6.5 µL)** | **Right reservoir (65 µL)** |
| --- | --- | --- |
| fMLP (60 µL) + autologous serum (540 µL) (10 %) | Gel:   - Medium: - 20 µL MEM (Minimum Essential Medium Eagle, Sigma-Aldrich Chemie GmbH, Steinheim, Deutschland) - 20 µL distilled water - 10 µL Sodium bicarbonate (Sodium bicarbonate solution, 7.5 %, Sigma-Aldrich Chemie GmbH, Steinheim, Deutschland) - 50 µL RPMI 1640 (PAN Biotech GmbH, Aidenbach, Deutschland) - 150 µL Collagen - 90 µL RPMI - 10 µL Serum - Colorants (DAPI, DHR, MPO each solved in RPMI/PBS without CaCl_2_/MgCl_2_) | Test series 1: substance effect  E2   \| Zero sample \| β-Estradiol [5 µM] \| β-Estradiol [0.01 µM] \| \| --- \| --- \| --- \|   BPA   \| Zero sample \| BPA [16 µM] \| BPA [1.6 µM] \| \| --- \| --- \| --- \| |
|  |  | Test series 2: centrifugation/centrifugation and substance effect  E2 and centrifugation   \| Zero sample \| Zero sample (centrifuged) \| β-Estradiol [5 µM] (centrifuged)  or  β-Estradiol [0.01] (centrifuged) \| \| --- \| --- \| --- \|   BPA and centrifugation   \| Zero sample \| Zero sample (centrifuged) \| BPA [16 µM] (centrifuged)  or  BPA [1.6 µM] (centrifuged) \| \| --- \| --- \| --- \| |

X [µm]

[µm]

Y [µm]

Y [µm]

Track Length

Track Displacement Length

Track Length

Supplementary Figure 2: Track Length and Track Displacement Length


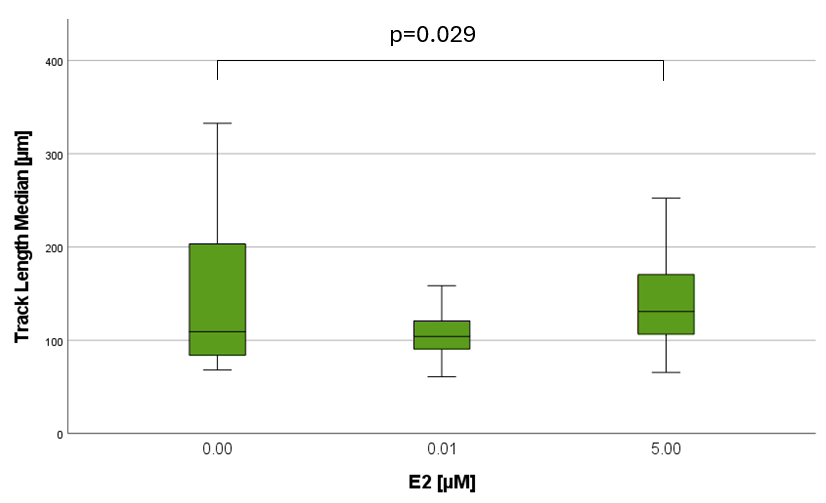


Supplementary Figure 3: The impact of E2 on TL - technical replicate analysis. Median TL is shown on the Y-axis. Analyzing six experiments with balanced sex ratio. In each case, 60 median TL are shown (β-Estradiol [5 µM]:59). TL median (IQR) values [µm]: Zero sample: 109 (121), β-Estradiol [0.01 µM]: 104 (34), β-Estradiol [5 µM]: 131 (65).


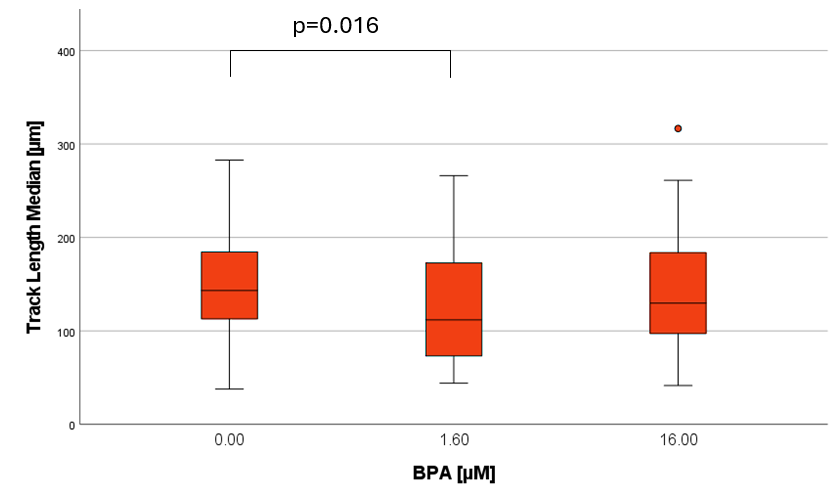


Supplementary Figure 4: The impact of BPA on TL - technical replicate analysis. Median TL is shown on the Y-axis. Analyzing six experiments with balanced sex ratio. In each case, 60 median TL are shown. While the Kruskal-Wallis test revealed no overall group differences (p= 0.051), an exploratory pairwise comparison indicated a significant difference between zero sample an BPA [1.6 µM]. TL median (IQR) values [µm]: Zero sample: 143 (74), BPA [1.6 µM]: 112 (100), BPA [16 µM]: 130 (88).

Supplementary Table 3: Overview of receptors and cellular signaling pathways modulated by BPA across different cell types (column 1). Receptors and signaling pathways in PMNs, which are described to be modulated by BPA and E2 (column 2 and 3).

| **BPA-responsive signaling pathways in various cell lines (23)** | **BPA-responsive signaling pathways in PMNs** | **E2-responsive signaling pathways in PMNs** |
| --- | --- | --- |
| Estrogen receptor (ERα and ERβ) signaling | Estrogen receptor (ERβ) signaling (15)  ↑ PI3K-Akt signaling pathway in male PMNs (14)  ↓ CXCL8/ IL-8 (46) | Estrogen receptor (ERα and ERβ) signaling (43) |
| Estrogen-related receptor ERRγ signaling |  |  |
| G-protein-coupled estrogen receptor (GPER) signaling |  | GPER1: cAMP/PKA/CREB; p38 MAPK; ERK1/2 (47) |
| Aryl hydrocarbon receptor (AhR) signaling |  |  |
| Androgen receptor (AR), Thyroid receptor (TR), Glucocorticoid receptor (GR) signaling |  |  |
| Peroxisome proliferator-activated receptors PPARα, y, β/δ signaling |  |  |
| Pregnane X receptor (PXR) signaling |  |  |
| TGF-β signaling |  |  |
| Epigenetic modifications |  |  |

Supplementary Table 4: The effect of E2 and BPA on migration - comparison projects. The down arrow (↓) indicates a decrease in migration. The upward-pointing arrow (↑) symbolizes an increase in migration behavior. No influence on migration is marked by the horizontal arrow (↔). Sex annotation: f=female, m=male.

| **Migration** | **Results** | **Sex results** | **Literature** | **Results** | | **Concentration** | **Sex** |
| --- | --- | --- | --- | --- | --- | --- | --- |
| β-Estradiol [0.01 µM] | ↓ | f: ↓  m: ↑ | (11) | | ↓ | 1 nM,  100 nM | m |
|  |  |  | (12) | | ↓ | 0.1 ^–^ 1000 nM  0.1 -1 nM | m, f |
|  |  |  | (14) | | ↔ | 0.1 nM | m, f |
| β-Estradiol [5 µM] | ↑ | f: ↑  m: ↑ |  | | | | |
| BPA [1.6 µM] | ↓ | f: ↓  m: ↓ | (15) | | ↓ | 0.1 µM, 1 µM | No indication of sex |
|  |  |  | (14) | | ↓ | 16 nM, 1.6 µM | m, f |
| BPA [16 µM] | ↓ | f: ↑    m: ↓ |  | | | | |

Supplementary Table 5: The effect of E2 and BPA on cell surface expression - comparison projects. The down arrow (↓) indicates a decrease in antigen expression. The upward-pointing arrow (↑) symbolizes an increase in antigen expression. No influence is marked by the horizontal arrow (↔). Sex annotation: f=female, m=male.

| **Cell surface antigens** | **Results** | **Sex**  **results** | **Literature** | **Results** | **Concentration** | **Sex** |
| --- | --- | --- | --- | --- | --- | --- |
| β-Estradiol [0.01 µM] | CD66b↑  CD11b↓ | f: CD66b ↑  CD11b ↓  m: -- | (8) | ↔ CD11b  CD33  CD34 | 10^-8^ g/mL | m, f |
|  |  |  | (14) | ↔ CD14  CD284 | 0.1 nM | m, f |
| β-Estradiol [5 µM] | CD11b↓ | f: CD11b ↓  m: -- |  | | | |
| BPA [1.6 µM] | ↑LOX1  ↓CD11b | f: LOX1 ↑  CD11b ↓  m: -- | (26) | ↑ CD14  ↓ CD11c  CD15  CD16 | 16 nM | f |
|  |  |  |  | ↑ CD14  ↓ CD15  CD16 | 16 nM | m |
| BPA [16 µM] | ↓CD11b | f: CD11b ↓  m: -- |  | ↓ CD11c  CD15  CD16 | 1.5 µM, 3 µM, 6 µM, 12 µM | f |
|  | | |  | ↓ CD15  CD16  ↑ CD62L | 1.5 µM, 3 µM, 6 µM, 12 µM  12 µM | m  f |
|  |  |  |  | ↓ CD11c | 12 µM | m |

Supplementary Table 6: The effect of E2 and BPA on oxidative burst - comparison projects. The down arrow (↓) indicates a decrease in oxidative burst. The upward-pointing arrow (↑) symbolizes an increase in oxidative burst. Sex annotation: f=female, m=male.

| **Oxidative Burst** | **Results** | **Sex results** | **Literature** | **Results** | **Concentration** | **Sex** |
| --- | --- | --- | --- | --- | --- | --- |
| β-Estradiol [0.01 µM] | ↑ PMA | f: ↑ PMA  m: ↑ PMA | (8) | ↑ (PMA) | 10^-12^ – 10^-7^ g/mL | f |
|  |  |  | (42) | ↓ (fMLP)  (reduction of  ferricytochrome-C) | 100 nM | m, f |
|  |  |  | (40) |  | 1-100 nM |  |
| β-Estradiol [5 µM] | ↑ PMA | f: ↑ PMA  m: -- | (37) | ↑  (G-6-PDase translocation) |  | pregnant women |
|  |  |  | (39) | reduced H_2_O_2_ formation |  |  |
| BPA [1.6 µM] | ↑ PMA | f: ↑ PMA  m: ↑ PMA | (15) | ↑  (dichlorofluorescin diacetate  DCFDA method) | 0.03-100 µM | m, f |
| BPA [16 µM] | -- | -- |  |  |  |  |
